# Osteoclast-Driven Polydopamine-to-Dopamine Release: An Upgrade Patch for Polydopamine-Functionalized Tissue Engineering Scaffolds

**DOI:** 10.3390/jfb15080211

**Published:** 2024-07-29

**Authors:** Lufei Wang, Huamin Hu, Ching-Chang Ko

**Affiliations:** 1 Guangxi Key Laboratory of Oral and Maxillofacial Rehabilitation and Reconstruction & College and Hospital of Stomatology, Guangxi Medical University, Nanning 530021, China; 2 Division of Oral and Craniofacial Health Sciences, Adams School of Dentistry, University of North Carolina, Chapel Hill, NC 27599, USA; 3 Division of Orthodontics, College of Dentistry, The Ohio State University, Columbus, OH 43210, USA

**Keywords:** bone tissue engineering, bone regeneration, drug release, dopamine, osteoclasts

## Abstract

Polydopamine, a mussel-inspired self-adherent polymer of dopamine, has impressive adhesive properties and thus is one of the most versatile approaches to functionalize tissue engineering scaffolds. To date, many types of polydopamine-functionalized scaffolds have been manufactured and extensively applied in bone tissue engineering at the preclinical stage. However, how polydopamine is biodegraded and metabolized during the bone healing process and the side effects of its metabolite remain largely unknown. These issues are often neglected in the modern manufacture of polydopamine-functionalized materials and restrict them from stepping forward to clinical applications. In this study, using our bioinspired polydopamine-laced hydroxyapatite collagen calcium silicate material as a representative of polydopamine-functionalized tissue engineering scaffolds, we discovered that polydopamine can be metabolized to dopamine specifically by osteoclasts, which we termed “osteoclast-driven polydopamine-to-dopamine release”. The released dopamine showed an osteoinductive effect in vitro and promoted bone regeneration in calvarial critical-sized defects. The concept of “osteoclast-driven polydopamine-to-dopamine release” has considerable application potential. It could be easily adopted by other existing polydopamine-functionalized scaffolds: just by recruiting osteoclasts. Once adopted, scaffolds will obtain a dopamine-releasing function, which enables their modulation of osteoblast activity and hence elevates the osteoinductive effect. Thus, “osteoclast-driven polydopamine-to-dopamine release” serves as an upgrade patch, which is useful for many existing polydopamine-functionalized materials.

## 1. Introduction

Bone defects are common and often result from infection, trauma, or tumors. Although bone graft is often recommended to restore bone integrity and function, the management of bone defects remains a challenge for clinicians nowadays, particularly when defects are large-sized [1,2]. The rapid development of tissue engineering technology makes it a promising strategy for repairing large bone defects. In tissue engineering, scaffolds serve as the extracellular matrix to support cell colonization and guide tissue regeneration at the defect site [3,4].

Polydopamine is a biopolymer derived from dopamine oxidation and self-polymerization, which is bioinspired by the adhesive nature of catechols and amines in mussel adhesive proteins. Due to its desirable properties, such as the simplicity of preparation, universal adhesion, good biocompatibility, and drug immobilization capacity, polydopamine has become one of the simplest and most versatile approaches to functionalize tissue engineering scaffolds. It can enhance a scaffold’s mechanical strength via cross-linking mechanisms, form a hydrophilic surface coating to improve a material’s biocompatibility and cell adhesion, or make the surface chemically active to immobilize therapeutic agents for drug delivery purposes [5,6,7,8]. To date, a variety of polydopamine-functionalized scaffolds have been manufactured, many of which perform very well in repairing large bone defects at the preclinical stage and may even exert immunomodulatory, anti-inflammatory, or anti-tumor effects [9,10,11,12]. For example, our in-house, bioinspired, polydopamine-laced hydroxyapatite collagen calcium silicate (PDHC) material showed good mechanical properties and significantly facilitated new bone formation in calvaria critical-sized defects [13,14,15].

During bone defect regeneration, the implanted scaffold is expected to degrade progressively, either by physicochemical dissolution or osteoclastic resorption, to spare space for new bone formation [16]. Loading osteoinductive agents, such as growth factors or small molecule compounds, into biodegradable scaffolds can achieve a sustained and local drug release throughout the healing process of bone defects, and thus they are often employed to facilitate bone defect regeneration [17,18,19]. However, how polydopamine is biodegraded and metabolized during the bone regeneration process and the side effects of its metabolite remain largely unknown. These issues are often neglected in the modern manufacture of polydopamine-functionalized materials and may restrict them from being used in clinical applications. Aiming to explore this issue, we herein used our previously published PDHC material which interacts well with osteoclasts (OCs) [15] as a representative of existing polydopamine-functionalized scaffolds, and accidentally discovered an “OC-driven polydopamine-to-dopamine release” phenomenon that may facilitate bone regeneration. This phenomenon might be universal and thus could be adopted by other polydopamine-functionalized materials as an upgrade patch.

## 2. Materials and Methods

### 2.1. Fabrication of PDHC Material

PDHC material was fabricated according to our previous method [13,14]. Briefly, wax molds for making 2D disks or 3D scaffolds were prepared using a Form 2 3D printer (Formlabs, Boston, MA, USA). The mold was 8.5 mm in diameter, 1.2 mm in thickness, and contained 500 μm pores (if making a 3D scaffold). Hydroxyapatite (Sigma-Aldrich, St. Louis, MO, USA, cat# 900203) and collagen (Sigma-Aldrich, St. Louis, MO, USA, cat# C7661) slurry was synthesized by a co-precipitation method using the in situ hybridization of calcium silicates (Sigma-Aldrich, St. Louis, MO, USA, cat# 742503) with hydroxyapatite–collagen powder [13]. The particle size of the synthesized hydroxyapatite crystals in the aqueous gel solution was mainly between 25–100 nm. The hydroxyapatite–collagen powder, calcium hydroxide (Sigma-Aldrich, St. Louis, MO, USA, cat# 1.02119), and dopamine (Sigma-Aldrich, St. Louis, MO, USA, cat# H8502) were mixed and cross-linked with enTMOS (bis [3-(trimethoxysilyl)-propyl] ethylenediamine) (Gelest Inc., Morrisville, PA, USA, cat# SIB1834.1) and ammonium persulfate (Sigma-Aldrich, St. Louis, MO, USA, cat# 248614) in the cold stage. Then, the mixture was poured into wax molds for solidification for about 20 min. After dewaxing, the product—PDHC disks—were immersed in 3% glycine for neutralization for a few days. Finally, the PDHC disks were air-dried, oven-dried, and sterilized with ethylene oxide gas for subsequent experimental use. Hydroxyapatite collagen calcium silicate (HCCS) material is the dopamine-free, analogue control for PDHC.

To validate physico-chemical characteristics, PDHC samples were ground to a powder and analyzed using a Nicolet 670 Fourier transform infrared (FTIR) spectroscope (Thermo Scientific, Waltham, MA, USA). The data showed the characteristic chemical bonding peaks, indicating the presence of hydroxyapatite gel, enTMOS, calcium silicate, polydopamine, etc. (Supplementary Figure S1A). X-ray diffraction (XRD) was also performed on PDHC powder using a Philips X-ray automated powder diffractometer equipped with a Cu target and a Ge post-sample monochromator. The data collection parameters were: 5° to 50°, 450 steps, 1 s per step. The data showed peaks representing the different phases of hydroxyapatite, Ca(OH)_2_, and calcium silicate (Supplementary Figure S1B). Overall, these data validated the successful fabrication of PDHC.

### 2.2. OC Differentiation

RAW 264.7 (ATCC^®^ TIB-71) cells were maintained in DMEM (Gibco) supplemented with 10% FBS (Gibco, Waltham, MA, USA) and penicillin/streptomycin at 37 °C, 5% CO_2_. For osteoclastogenic induction, RAW cells were seeded on PDHC disks at a density of 4 × 10^4^ cells/cm^2^ (so-called 1X) or 8 × 10^4^ cells/cm^2^ (so-called 2X) and treated with 10 ng/mL receptor activator of nuclear factor kappa-Β ligand (RANKL) (R&D systems, Minneapolis, MN, USA) for up to 12 days. The medium was replenished every two days. OC-conditioned medium was collected at indicated time points (4 d, 8 d, 12 d), and incorporated into the medium for treating mesenchymal stem cells (MSCs) at a 1:1 ratio.

For OC visualization, cells were seeded on PDHC-coated tissue culture dishes. OCs were fixed using 4% paraformaldehyde, permeabilized by Triton X-100, and stained using a tartrate-resistant acid phosphatase (TRAP) staining kit (Sigma-Aldrich, St. Louis, MO, USA, cat# 387A). TRAP-positive multinucleated cells were identified as OCs under bright field imaging using a Nikon Eclipse Ti-U inverted microscope (Nikon, Tokyo, Japan).

Primary OC culture was also performed. Bone marrow cells were flushed out from mouse femurs (8-week-old animals, C57BL/6J) into the same medium as RAW cells. Non-adherent cells were collected overnight, re-plated on PDHC disks at a density of 1.5 × 10^5^ cells/cm^2^, and treated with 30 ng/mL M-CSF (R&D systems, Minneapolis, MN, USA) for 3 days to enrich monocytes. Then, cells were treated with 30 ng/mL M-CSF and 10 ng/mL RANKL (R&D systems, Minneapolis, MN, USA) for 4 days for OC differentiation.

### 2.3. Assessment of Osteoclastic Degradation of PDHC Material

For PDHC degradation percentage measurement, the initial weight of each PDHC disk was recorded. After 4 d, 8 d, and 12 d of interaction with OCs, PDHC disks were removed from the medium, washed with distilled water, dried, and weighed. The percentage of weight loss was calculated.

Next, the PDHC disks were sonicated and scratched using a soft brush to remove cells. The disks were fixed in 10% formalin, washed, and underwent critical point drying. Then, the disks were sputter-coated and imaged using a Hitachi S-4700 scanning electron microscope (SEM) (Hitachi High-Tech, Tokyo, Japan).

### 2.4. Dopamine Concentration Measurement

Dopamine concentration was determined using a highly sensitive dopamine ELISA kit (Sangon Biotech, Shanghai, China, cat# D751019). OC-conditioned medium (collected from 1 d to 12 d) and tissue fluid samples (collected at 2, 4, 6, and 8 weeks post-surgery) were incubated with a dopamine antibody pre-coated plate. After being sequentially treated with the detection antibody, HRP-conjugated streptavidin, and substrate, the absorbance of the samples was read at 450 nm.

### 2.5. Gene Knockdown

RAW cells were seeded on PDHC disks at a density of 4 × 10^4^ cells/cm^2^ and were transfected with siRNAs using Lipofectamine RNAiMAX (Invitrogen, Waltham, MA, USA, cat# 13778) to knockdown target genes. The siRNAs used include: *Tcirg1* (ThermoFisher, Waltham, MA, USA, siRNA# 188671), *Ctsk* (ThermoFisher, Waltham, MA, USA, siRNA# 160424), *Mmp9* (ThermoFisher, Waltham, MA, USA, siRNA# 156835), and *Rab5a* (ThermoFisher, Waltham, MA, USA, siRNA# 152489). At 24 h post transfection, cells were washed off and started OC induction. Dopamine concentration in conditioned medium was detected on day 12.

### 2.6. MSC Culture and Osteogenesis Evaluation

Bone marrow-derived MSCs were isolated from 8-week-old C57BL/6J mice in accordance with the ARRIVE guidelines and a protocol of the IACUC at Ohio State University (No. 2020A00000064). After euthanasia, femurs were removed and bone marrow was then flushed out and maintained in DMEM (Gibco, Waltham, MA, USA) supplemented with 10% FBS and penicillin/streptomycin. Floating cells were removed overnight and attached cells were kept for expansion. The MSCs in passages 3–5 were verified using flow cytometry (positive for CD29, CD44, and CD105 and negative for CD34 and CD45) for subsequent use. For osteogenic induction, an osteogenic medium, which is the complete growth medium supplemented with 10 mM β-glycerophosphate, 0.2 mM ascorbic acid, and 10^−7^ M dexamethasone, was applied to MSC for 14 days.

MSC viability at 24 h, 48 h, and 96 h were detected by the RealTime-Glo™ MT cell viability assay (Promega, Madison, WI, USA) following the manufacturer’s protocol. Cell viability was represented as the luminescence value, measured in relative light units (RLUs).

For evaluating alkaline phosphatase (ALP) activity, MSC lysates were assayed using a colorimetric ALP assay kit (Abcam, Cambridge, UK, cat# ab83369). Absorbance was read at 405 nm.

Osteogenic gene (*Osx* and *Ocn*) expression was detected by RT-qPCR. After total RNA extraction and cDNA reverse-transcription, RT-qPCR was performed on the StepOnePlus Real-time PCR system (Applied Biosystems, Waltham, MA, USA) using the iTaq™ Universal SYBR Green Supermix (Bio-Rad, Hercules, CA, USA). The expression of target genes was normalized to the housekeeping gene β-2-microglobulin (*B2m*) and calculated by the ΔΔCT method. Primer sequences (5′-3′): ① *Osx*: forward CTCGTCTGACTGCCTGCCTAG, reverse GCGTGGATGCCTGCCTTGTA. ② *Ocn*: forward CCGGGAGCAGTGTGAGCTTA, reverse AGGCGGTCTTCAAGCCATACT. ③ *B2m*: forward TTCTGGTGCTTGTCTCACTGA, reverse CAGTATGTTCGGCTTCCCATTC.

### 2.7. Calvarial Critical-Sized Defect Model

A calvarial critical-sized defect was performed on 12-week-old, male Sprague Dawley rats in accordance with the ARRIVE guidelines and a protocol of the IACUC at the University of North Carolina at Chapel Hill (No. 18-201). Three groups (n = 4) were planned: HCCS scaffold, PDHC scaffold, and PDHC scaffold, seeded with differentiated OC. The surgical procedure has been described previously [14]. In brief, after general anesthesia, an 8 mm diameter defect was created at the center of the cranium using a trephine bur. After inserting the scaffold into the defect, Gelfoam^®^ was placed around the defect edge and a titanium mesh was placed between the periosteum and skin above the defect site to prevent scaffold micromovement. Pain management and anti-infectives were carried out post-surgery. Subcutaneous tissue fluid was collected from the defect site at indicated time points for a dopamine concentration assay. The rats were euthanized 8 weeks post-surgery.

### 2.8. MicroCT Analysis

After animal euthanasia, calvarias were harvested, fixed in 10% formalin, and scanned by a Skyscan 1076 microCT (Bruker, Billerica, MA, USA). The scanning parameters were: 40 kV, 1000 mA, 20 μm resolution, and 720 ms integration time. The 3D reconstruction was performed using ITK-SNAP software. New bone formation in the defect area was quantified using the Geomagic Design X 2020.0.4 software (3D Systems, Rock Hill, SC, USA).

### 2.9. Statistical Analyses

All experiments were performed in triplicate and repeated three times unless otherwise noted; data were plotted as mean ± SEM. The following statistical analyses were performed using GraphPad Prism 10.0.2 (GraphPad Software, San Diego, CA, USA): Student’s *t* test for the comparisons between two groups; one-way ANOVA for multiple groups comparisons. *p* < 0.05 indicates significance.

## 3. Results and Discussion

### 3.1. PDHC Releases Dopamine Specifically upon Osteoclastic Resorption

**Figure 1:**
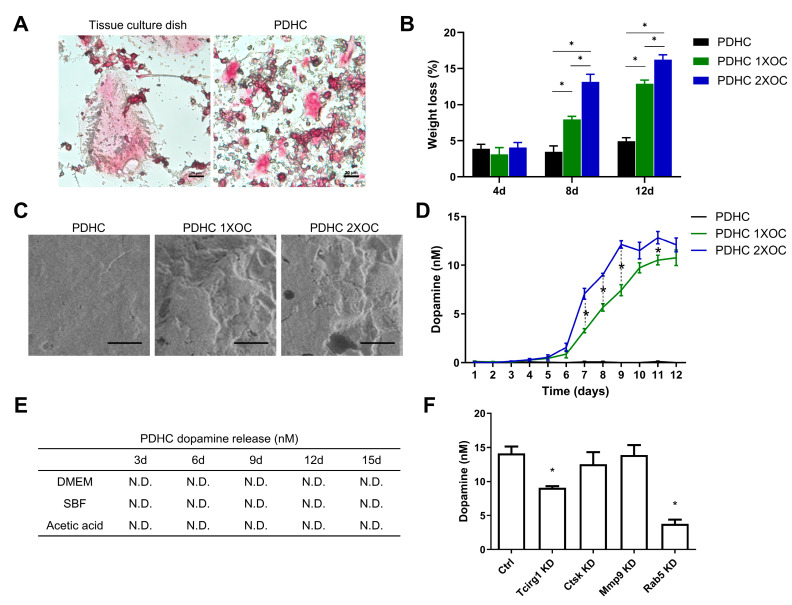
For (**A**–**D**), RAW cells were differentiated to OCs on PDHC disks. “1X OC” indicates 4 × 10^4^ cells/cm^2^ and “2X OC” indicates 8 × 10^4^ cells/cm^2^. (**A**) OC morphology on PDHC coating, visualized by TRAP staining. Scale bar is 20 µm. (**B**) PDHC material degradation percentage during osteoclastic resorption. * *p* < 0.05. (**C**) SEM images of PDHC surface after osteoclastic resorption. Images are shown on 12 d and the scale bar is 200 µm. (**D**) dopamine release curve of PDHC-OC constructs. * *p* < 0.05 when “PDHC 1X OC” vs. “PDHC 2X OC”. (**E**) dopamine release when PDHC immersed in various solutions. N.D.: not detected. (**F**) dopamine release from PDHC-OC constructs after knocking down target genes. Time point: day 12. * *p* < 0.05 compared to the Ctrl group.

OCs can be successfully differentiated on the PDHC surface, although the cell size is smaller (Figure 1A). During PDHC-OC interactions, PDHC can be degraded and resorbed by OCs progressively, and a higher number of OCs resulted in more erosion of PDHC (Figure 1B,C). Very intriguingly, a sustained release of dopamine (not polydopamine) was detected only upon osteoclastic resorption, particularly after day 6 when OCs usually become mature and fully functional, suggesting an OC-triggered polydopamine-to-dopamine conversion (Figure 1D). Additionally, this dopamine release seems responsive to OC number: 2X OC number (8 × 10^4^ cells/cm^2^) showed more dopamine release than 1X (4 × 10^4^ cells/cm^2^), which may be because more OCs can degrade PDHC faster. To confirm the indispensability of OCs for polydopamine-to-dopamine release, we noticed that the PDHC disk alone (no OCs presence) did not release dopamine when immersed in various solutions (Figure 1E). So, what molecule or signaling pathway within OCs is possibly responsible for this? So far, there is no report about how OCs can convert polydopamine to dopamine. “Bone eater” OCs possess comprehensive bone-resorbing machinery, such as vacuolar-ATPases that pump protons to dissolve hydroxyapatite, and proteolytic enzymes (Ctsk, Mmp9, etc.) for degrading collagenous bone matrix [20]. Among these activities, considering that acid alone was unable to make PDHC release dopamine (Figure 1E), we speculated that the OC-driven polydopamine-to-dopamine conversion was more likely attributed to OC enzymatic activities rather than acidic dissolution. We further conducted a gene silencing assay and found that osteoclastic acid production (Tcirg1) and the endocytosis pathway (Rab5) contributed to this polydopamine-to-dopamine conversion (Figure 1F), which may offer a direction for future investigations.

In our opinion, this OC-driven polydopamine-to-dopamine release feature has at least two meanings. On one hand, it may be cautionary for the clinical application of many existing polydopamine-functionalized tissue engineering scaffolds, because they will inevitably make contact with OCs in vivo and thus release bioactive dopamine unwantedly that may cause side effects. On the other hand, if the released dopamine can be well-utilized or well-manipulated, OC-driven polydopamine-to-dopamine release could be an upgrade patch for many existing polydopamine-functionalized scaffolds by unlocking a dopamine-releasing function. In contrast to polydopamine, we and other scholars have previously demonstrated that dopamine is an osteogenic biomolecule that can modulate a set of activities in osteoblast lineage cells, including adhesion, osteogenic differentiation, and mineralization, which are overall beneficial for bone regeneration [21,22,23]. If we imagine that polydopamine optimizes a material’s performance during scaffold manufacturing while dopamine stimulates bone formation during bone defect healing, this polydopamine-to-dopamine conversion may enable a “one drug, different use over time” phenomenon in modern tissue engineering.

### 3.2. PDHC-OC Interactions Release Dopamine That Shows Osteoinductive Activity

**Figure 2:**
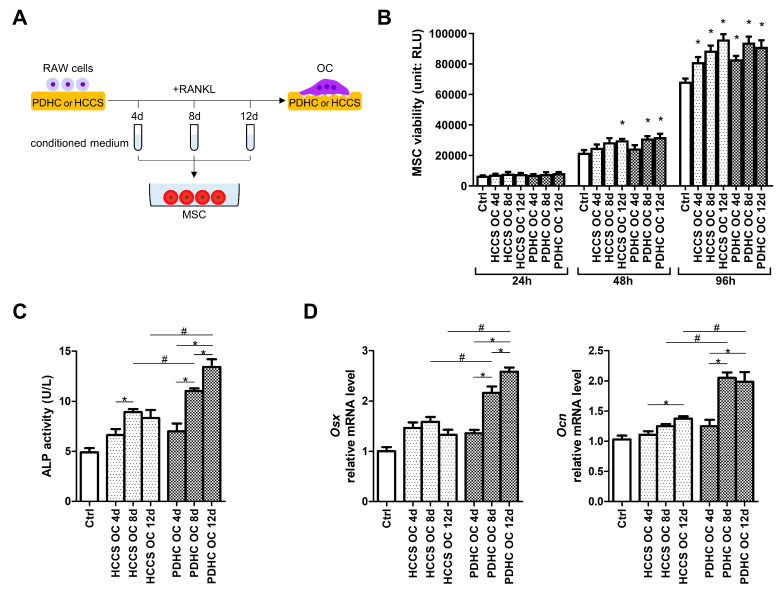
(**A**) Experiment design for (**B**–**D**): collect the conditioned medium of PDHC-OC or HCCS-OC construct at different time points and test their influences on MSC. (**B**) Effect on MSC viability. * *p* < 0.05 compared to the matched time point “Ctrl” group. (**C**,**D**) Effect on MSC ALP, *Osx*, and *Ocn* expression. * *p* < 0.05, # *p* < 0.05.

Next, we checked whether the dopamine released from PDHC-OC interactions possess osteoinductive activity through the following assays. We collected the conditioned medium at various stages (4 d, 8 d, 12 d) of PDHC-OC interaction, which should contain released dopamine, and checked their impact on MSC osteogenic activity (Figure 2A). HCCS material is the dopamine-free analogue control for PDHC. We noted that neither the conditioned mediums of the PDHC-OCs nor the HCCS-OCs impaired MSC viability (Figure 2B), and no difference was observed between the conditioned medium treatment of PDHC-OCs and HCCS-OCs, indicating that the dopamine contained in the PDHC-OC conditioned medium did not alter MSC viability.

After 14 days of osteogenic induction, most of the conditioned medium groups enhanced MSC osteogenic activity compared to the Control (“Ctrl”) group (Figure 2C,D), probably because OC itself can secrete a group of cytokines and growth factors termed “coupling signals” to stimulate osteoblast lineage cell activity [24,25,26]. PDHC-OC conditioned medium exhibited stronger osteogenic effects than HCCS-OC if the conditioned medium was collected at 8 d or 12 d, but not at 4 d (Figure 2C,D). This may be because dopamine release level from PDHC-OC on 4 d was too low to influence MSCs but reached sufficient dosage after 8 d (Figure 1D). This further explains the findings in our previous paper [15]. In that paper, although PDHC altered OC clastokine profile, PDHC-OC conditioned medium was collected only on 5d when the dopamine level may be too low, and so it overall did not alter MSC activity. In addition, the osteogenic power of PDHC-OC conditioned medium seems sorted as 4 d < 8 d < 12 d (Figure 2C,D), which is coincident with the growth of dopamine release over time (Figure 1D). Taken together, we conclude that the dopamine released from PDHC-OC interactions possess osteoinductive activity.

### 3.3. Manipulating Dopamine Release via Manipulating OC Number Influences PDHC’s Osteoinductive Effects

**Figure 3:**
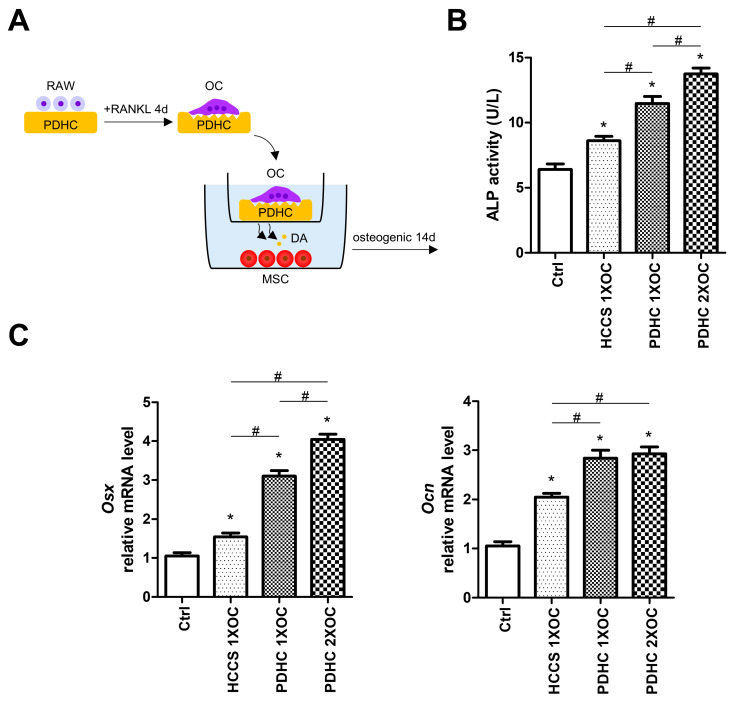
(**A**) Schematic diagram of the study. (**B**,**C**) After 14d osteogenic induction under Transwell co-culture condition, MSC ALP activity and expression of osteogenic genes, *Osx* and *Ocn*. * *p* < 0.05 compared to the Ctrl group. # *p* < 0.05.

Since we have shown that the dopamine released from PDHC-OC interaction seems responsive to OC number (Figure 1D), we next attempted to manipulate dopamine release via manipulating OC number (1X, 2X) and evaluated its osteoinductive effects in a Transwell co-culture system (Figure 3A). A PDHC-OC construct was placed in the upper chamber so it can provide a prolonged release of dopamine to MSCs. After 14 d of co-culture in the osteogenic medium, both PDHC-1X OC and PDHC-2X OC groups showed greater osteoinductive effects on MSCs compared to the Ctrl or HCCS-1X OC groups (Figure 3B,C). PDHC-2X OC displayed higher ALP activity and *Osx* expression level than the PDHC-1X OC group, indicating that seeding more OCs on the PDHC disk caused the greater release of dopamine and thus exerted greater osteoinductive effects. In addition, the above results have also been validated using murine primary OCs, which were differentiated from bone marrow-derived monocytes (Supplementary Figure S2).

These results enrich the operability of this OC-driven polydopamine-to-dopamine release system, although it is still in a very preliminary stage. Of course, there is a long way to go before it can be built into a mature drug release system. Some technical conundrums need to be improved. First, although the “OC-driven polydopamine-to-dopamine release” seems to be OC-dependent, as demonstrated by our in vitro experiments, how to exclude unintended activation by other cellular or biochemical processes within in vivo environments needs to be further investigated, as there are many other cell types present in the bone microenvironment. Second, controlling dopamine release via manipulating OC number is effective but somewhat rough. Excessive dopamine release should be prevented because it may cause systemic effects in vivo. We are seeking a more precise way to regulate the rate of dopamine release. Third, besides manipulating OC number, developing other means to adjust the dopamine release rate to better serve the varying needs of bone healing is recommended.

### 3.4. PDHC Provides a Sustained Release of Dopamine That Facilitates Calvaria Defect Repair

**Figure 4:**
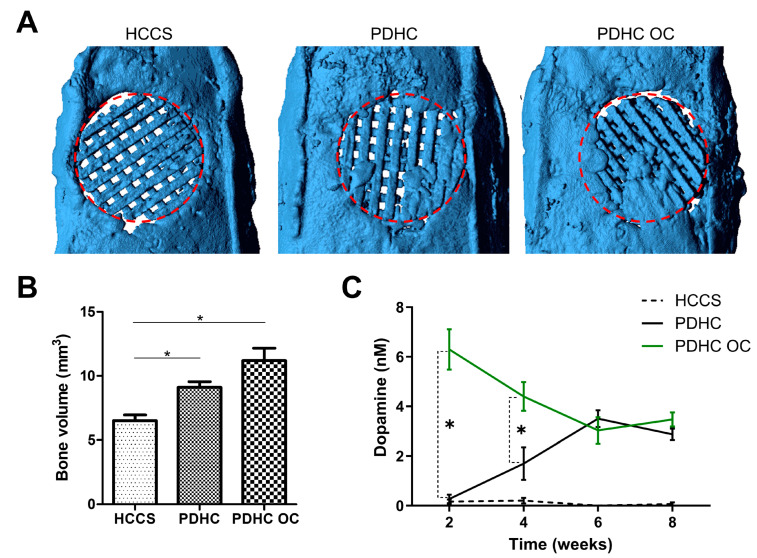
HCCS scaffold, PDHC scaffold, and exogenous-OC-seeded PDHC scaffold were implanted to repair rat calvarial critical-sized defects. Experiment period was 8 weeks; animal n = 4. (**A**) MicroCT images. The 8 mm in diameter red circle represents the defect site. (**B**) Quantification of new bone formation within the defect site. * *p* < 0.05. (**C**) Dopamine concentration in the tissue fluid from the defect site. * *p* < 0.05 when “PDHC” vs. “PDHC OC”.

After validating the osteoinductive effect of this OC-driven polydopamine-to-dopamine release system in vitro, we moved on to test it in a rat calvarial critical-sized defect model. PDHC scaffolds were implanted into the defect site and were anticipated to interact with endogenous OCs, hence releasing dopamine. HCCS scaffolds are the dopamine-free analogue control for PDHC. We also want to try manipulating dopamine release, so we seeded exogenous OCs into the PDHC scaffold as a “PDHC OC” group. During the 8 weeks post-surgery, very low levels of dopamine were detected in HCCS implantation, which may represent the physiological level of dopamine at the defect site (Figure 4C). Dopamine release in the PDHC group was successfully detected and seems significantly greater than physiological levels (represented by the HCCS control group), confirming the concern that polydopamine-functionalized scaffolds do release dopamine in vivo due to OC interactions (Figure 4C). During the bone defect healing process, endogenous OCs infiltrate and begin to resorb the scaffold, usually at a later stage of bone healing [27], which may explain why dopamine release increased as time increased. By contrast, loading exogenous OCs (“PDHC OC” group) can accelerate dopamine release, especially at the beginning (Figure 4C). As a result, at least partly attributed to the increased dopamine release, “PDHC OC” implantation showed much greater new bone formation than HCCS group and also tends to be greater than the PDHC group (although not statistically significant) (Figure 4A,B). This demonstrates that if well-manipulated, this OC-driven polydopamine-to-dopamine release could benefit bone defect repair and sometimes even expand the original utility of the scaffold, which is worth exploring in future studies.

Our previous study already showed that PDHC scaffolds can effectively promote new bone formation in critical-sized bone defects [14]. Now, the addition of the present polydopamine-to-dopamine release property upgraded PDHC scaffolds and improved their osteoconductive capacity in bone defect repair. It is possible that other existing polydopamine-functionalized tissue engineering scaffolds may acquire an extra dopamine-releasing function in this way to achieve broader application, which is worth a try in future studies. Of course, the present study is just a pilot study that foremost aims to convey a concept. Comprehensive in vitro and in vivo experiments are needed to further support the “OC-driven polydopamine-to-dopamine release” phenomenon before its large-scale application. Molecular biology assays or subcellular imaging technologies will be helpful to uncover the underlying cellular mechanisms of the OC-driven polydopamine-to-dopamine release.

## 4. Conclusions

**Figure 5:**
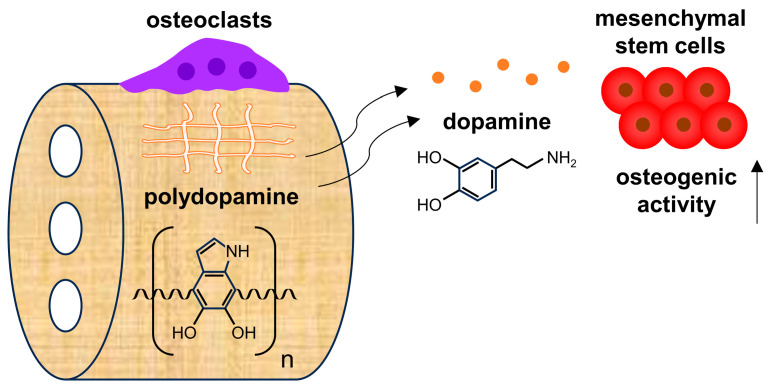
Concept map of OC-driven polydopamine-to-dopamine release.

In summary, using PDHC as an example of polydopamine-functionalized tissue engineering scaffolds, we preliminarily identified an OC-driven polydopamine-to-dopamine release feature that has considerable application potential (Figure 5): ① Rather than being cautionary for clinical applications, if well-utilized, it could be an upgrade patch for many existing polydopamine-functionalized scaffolds by unlocking a dopamine-releasing function. Polydopamine-to-dopamine conversion enables a “one drug, different use over time” phenomenon in modern tissue engineering. ② It can be very easily installed on other polydopamine-functionalized scaffolds—just by recruiting OCs. ③ It will have specificity to some extent; this polydopamine-to-dopamine conversion is OC-dependent.

## Data Availability

The original contributions presented in the study are included in the article, further inquiries can be directed to the corresponding authors.

## Supplementary Materials

The following supporting information can be downloaded at: https://www.mdpi.com/article/10.3390/jfb15080211/s1; Figure S1. (A) FTIR spectrum of PDHC material; (B) XRD measurement of PDHC material. Figure S2. Use bone marrow-derived monocytes instead of RAW cells as OC precursor for validation.
