# Supplementary material for: Osteoclast-Driven Polydopamine-to-Dopamine Release: An Upgrade Patch for Polydopamine-Functionalized Tissue Engineering Scaffolds
Source: J Funct Biomater. 2024 Jul 29;15(8):211. doi: 10.3390/jfb15080211 (PMC11355103; doi:10.3390/jfb15080211)
Supplement: Supplementary file 1 [file jfb-15-00211-s001.zip › jfb-3066252-supplementary.pdf]

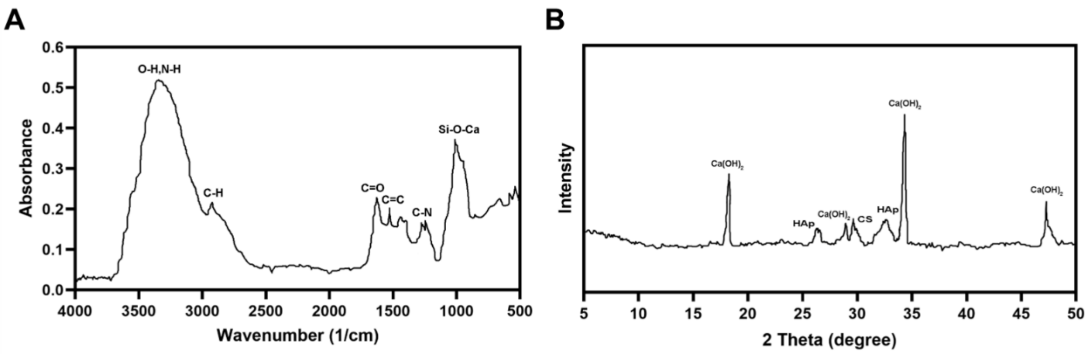

Supplementary Fig. S1. (A) FTIR spectrum of PDHC material. It contains the chemical bonding characteristic peaks indicating hydroxyapatite-gel, enTMOS, calcium silicate, polydopamine, etc. **(B)** XRD measurement of PDHC material. The peaks represent different phases of hydroxyapatite (HAp), $Ca(OH)_2$ and calcium silicate (CS).

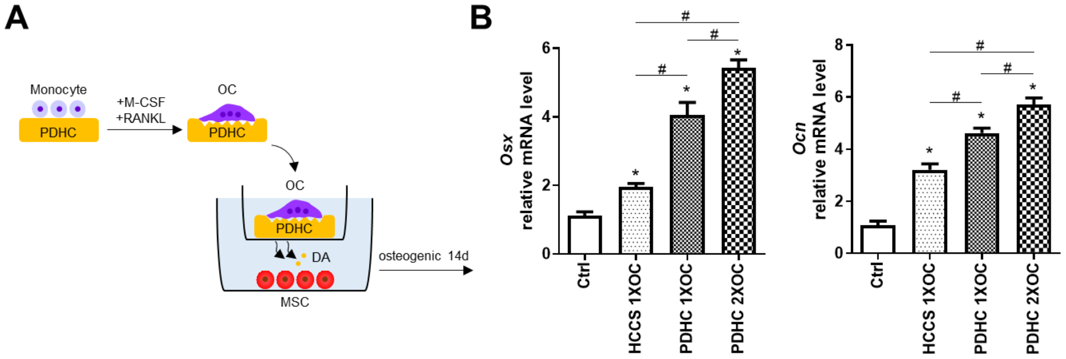

Supplementary Fig. S2. Use bone marrow-derived monocytes instead of RAW cells as OC precursor for validation. **(A)** Schematic diagram of the study. **(B)** After 14d osteogenic induction under transwell co-culture condition, MSC's osteogenic genes *Osx* and *Ocn* expression. *$P < 0.05$ compared to the Ctrl group. #$P < 0.05$.
